# 

**Published:** 1918-02-23

**Authors:** 


Rates of Subscription: Twelve months, 6/6; Six months, 4/-; three months, 2/-, post free to any address in the United Kingdom. Abroad, Twelve months, 8/8; Six months, 5/-; Three months, 2/6, post free. **THE HOSPITAL "Index" to Volume LXII., April 1917 to September 1917, is now ready, price 2½d. per copy, post free.** Address The Manager, The Hospital, "The Hospital" Building, 28/29 Southampton Street, Strand, London, W.C. 2.

### Annual Meetings.

Queen Mary's Hospital for the East End (Incorporated), Stratford, E.—In the Council Chamber, Town Hall, Stratford, on Saturday, February 23, at 3 p.m., C. E. Leo Lyle, Esq., presiding.

Central Council for District Nursing in London.—In the Conference Hall at the Local Government Board, Whitehall, S.W., on Tuesday, February 26, at 2.30 p.m. The Rt. Hon. W. Hayes Fisher, M.P., President of the Local Government Board, will receive the newly elected Council.

### Matrons' and Sisters' Appointments.

AUXILIARY RED CROSS HOSPITAL, BOWDON, CHESHIRE.—Sister Ault has been appointed sister-in-charge. She was trained at the Clayton Hospital and General Dispensary, Wakefield, has been sister at the Victoria Hospital, Tynemouth, the Stratford-on-Avon Hospital, and Princess Alice Hospital, Eastbourne, and matron of the Westmorland Sanatorium, Meathop, Grange-over-Sands, and the Meols Hall Auxiliary Hospital, Southport.

BOOTH HALL INFIRMARY, MANCHESTER.—Miss Margaret Cave has been appointed first assistant matron. She was trained at St. Pancras Infirmary South, and was later staff nurse at St. Giles' Infirmary, Camberwell, ward sister at St. Saviour's Infirmary, East Dulwich, and at Whipps Cross Infirmary, Leytonstone. She has been second assistant matron at Booth Hall Infirmary.

ESSEX COUNTY HOSPITAL, COLCHESTER.—Miss Mary E. Jones has been appointed matron. She was trained at Guy's and Brompton Hospitals, has been night superintendent and ward sister at Manchester Royal Eye Hospital, district nurse at Guy's Hospital, sister at York County Hospital, and assistant matron at the Ecole Belge d'Infirmières Diplomées, Brussels, at the Devonshire Hospital, Buxton, and at the Hampstead Military Hospital.

ROCHDALE INFIRMARY, ROCHDALE.—Miss Helena M. Ridgway has been appointed matron. She was trained at the Royal Infirmary, Preston, and has been sister-in-charge of the female surgical wards, night superintendent and assistant matron at the Blackburn and East Lancashire Royal Infirmary.

ST. BARTHOLOMEW'S HOSPITAL, ROCHESTER.—Miss Grace Farquhar has been appointed home sister and assistant matron. She was trained at St. Bartholomew's Hospital, London, has been night sister at Bromley Hospital, night sister and ward sister at St. Bartholomew's Hospital, Rochester, and sister at Birmingham General Hospital.

ST. MARY, ISLINGTON, INFIRMARY, HIGHGATE.—Miss F. C. Wallen, A.R.R.C., has been appointed matron. She was trained at the Metropolitan Hospital, has been ward sister and night superintendent at Southwark Infirmary, home sister at Fulham Infirmary, and assistant matron at St. Mary, Islington, Infirmary. Miss Winifred Shambrook and Miss Ellen Worvell have been appointed sisters. They were trained at the above institution.

**NOTE.—Particulars of Appointments for insertion in this column will be welcomed.**

